# Renal phenotyping in a hypomorphic murine model of propionic aciduria reveals common pathomechanisms in organic acidurias

**DOI:** 10.1038/s41598-024-79572-z

**Published:** 2024-12-16

**Authors:** Anke Schumann, Ainhoa Martinez-Pizarro, Eva Richard, Christoph Schell, Anna Laura Kössinger, Karina A. Zeyer, Stefan Tholen, Oliver Schilling, Michael Barry, Björn Neubauer, Michael Köttgen, Luciana Hannibal, Lourdes R. Desviat, Ute Spiekerkötter

**Affiliations:** 1 Department of General Paediatrics, Adolescent Medicine and Neonatology, Faculty of Medicine, Medical Center, University of Freiburg, Breisacherstr. 62, 79106 Freiburg, Germany; 2 Centro de Biología Molecular Severo Ochoa, UAM-CSIC, CIBERER, IdiPaz, IUBM, Universidad Autónoma de Madrid, Madrid, Spain; 3 Faculty of Medicine, Medical Center, University of Freiburg, Institute for Surgical Pathology, Freiburg, Germany; 4 Department of Medicine, Division of Infectious Diseases, Mayo Clinic, Rochester, MN 55905 USA; 5 Department of Medicine IV - Nephrology and Primary Care, Faculty of Medicine and Medical Center, University of Freiburg, Freiburg, Germany; 6 CIBSS - Centre for Integrative Biological Signaling Studies, University of Freiburg, Freiburg, Germany; 7 Department of General Paediatrics, Adolescent Medicine and Neonatology, Laboratory of Clinical Biochemistry and Metabolism, Faculty of Medicine, Medical Center, University of Freiburg, Freiburg, Germany

**Keywords:** Propionic aciduria, Mitochondrial dysfunction, Mitochondrial homeostasis, Mitochondrial fission, Chronic kidney disease, Mitochondrial energy metabolism, Mitochondrial quality control, Medical research, Molecular medicine

## Abstract

Mutations in the mitochondrial enzyme propionyl-CoA carboxylase (PCC) cause propionic aciduria (PA). Chronic kidney disease (CKD) is a known long-term complication. However, good metabolic control and standard therapy fail to prevent CKD. The pathophysiological mechanisms of CKD are unclear. We investigated the renal phenotype of a hypomorphic murine PA model (*Pcca*^*-/-*^*(A138T)*) to identify CKD-driving mechanisms. *Pcca*^*-/-*^*(A138T)* mice show elevated retention parameters and express markers of kidney damage progressing with time. Morphological assessment of the *Pcca*^*-/-*^*(A138T)* mouse kidneys indicated partial flattening of tubular epithelial cells and focal tubular-cystic dilation. We observed altered renal mitochondrial ultrastructure and mechanisms acting against oxidative stress were active. LC–MS/MS analysis confirmed disease-specific metabolic signatures and revealed disturbances in mitochondrial energy generation via the TCA cycle. Our investigations revealed altered mitochondrial networks shifted towards fission and a marked reduction of mitophagy. We observed a steep reduction of PGC-1-α, the key mediator modulating mitochondrial functions and a counter actor of mitochondrial fission. Our results suggest that impairment of mitochondrial homeostasis and quality control are involved in CKD development in PA. Therapeutic targeting of the identified pathways might help to ameliorate CKD in addition to the current treatment strategies.

## Introduction

Propionic aciduria (PA, OMIM # 606054) is caused by mutations in the mitochondrial enzyme propionyl-CoA carboxylase (PCC) which catalyzes the final steps of propionate metabolism. Elevated concentrations of propionylcarnitine (C3), 3-OH propionate and methylcitrate are biochemical hallmarks and may have toxic and signaling properties^1^. Despite ubiquitous PCC expression, long-term complications include cardiac and neurological impairment as well as chronic kidney disease (CKD), which points towards tissue-specific vulnerability.

Studies on the natural history of CKD in PA are scarce but highlight that CKD in PA occurs already in childhood^2,3^ and 50% of adult patients have a GFR of < 60 mL/min^4^. CKD in PA lacks a characteristic renal pattern and standard diagnostic markers like creatinine are poor predictors for onset and follow-up due to decreased muscle mass and low protein diet in PA patients^1^. A kidney biopsy in an adult PA patient revealed flattened tubular epithelium and cystic structures with mild focal lymphocytic inflammatory infiltrates^5^. Neither good adherence to diet nor liver transplantation prevents kidney function decline in PA^2,3^. CKD is a highly relevant but mechanistically not understood complication of PA.

Of note, CKD is a long-known complication in closely related methylmalonic aciduria (MMA), an organic aciduria (OA) presenting with severe and early onset CKD inevitably leading to end stage renal disease^6^. Methylmalonyl-CoA mutase lies two steps downstream of PCC. The metabolic profile is similar with the exception of high concentrations of methylmalonic acid. MMA is associated with tubulointerstitial nephritis and renal tubular acidosis^1^. The identification of similarities and differences in these metabolically closely related yet in terms of CKD severity different OAs might be helpful to untangle the underlying mechanisms.

Recently, different studies shed new light on potential CKD driving mechanisms in OAs. Our group and others^7–9^ identified mitochondrial alterations and oxidative stress (ROS) as potential disease driving mechanisms in both OAs. Gallego-Villar et al^10^ reported elevated ROS levels in PA patients´ fibroblasts and in different organs (brain, muscle, heart, liver) of the *Pcca*^*-/-*^*(A138T)* mouse model, and detected decreased oxidative phosphorylation (OXPHOS) capacity. Measurements of respiratory chain activity in the tissues of both PA and MMA patients showed relevant deficiencies^11^. The interference of toxic metabolites targeting mitochondrial proteins and leading to energetic dysfunction have been discussed for both entities^12^. Different renal MMA models revealed disturbance of mitochondrial homeostasis and mitochondrial quality control (MQC)^13^. Investigation of a renal epithelial cellular model for PA pointed in a similar direction. Cellular stress induced by high protein load aggravated the phenotype^8^. Involvement of posttranslational modification mechanisms like lysine acylation and methylmalonylation might explain altered enzymatic expression patterns and concomitantly function of distinct metabolic pathways^12,14^.

Comprehensive phenotypic examination and in depth molecular and metabolic profiling in tissue specific models are needed to better understand and treat CKD driving mechanisms in PA, particularly since human kidney is not easily accessible.

In this study, we explored potential CKD driving pathomechanisms in the hypomorphic *Pcca*^*-/-*^*(A138T)* mouse model. We hypothesized that OAs presenting with a renal phenotype might share specific patterns, which can be directly targeted in the future. In line with previous findings for PA and MMA, mitochondrial networks were abnormal in *Pcca*^*-/-*^*(A138T)* mice, while mechanisms counteracting ROS were active. We identified severe disturbances of mitochondrial homeostasis and dynamics while MQC was dysfunctional. The study highlights the role of the mitochondrion for CKD progression in PA and supports the idea that therapies targeting mitochondrial homeostasis might be renoprotective in OAs.

## Results

### Altered renal and mitochondrial function in *Pcca*^*-/-*^*(A138T)* mice

**Fig. 1:**
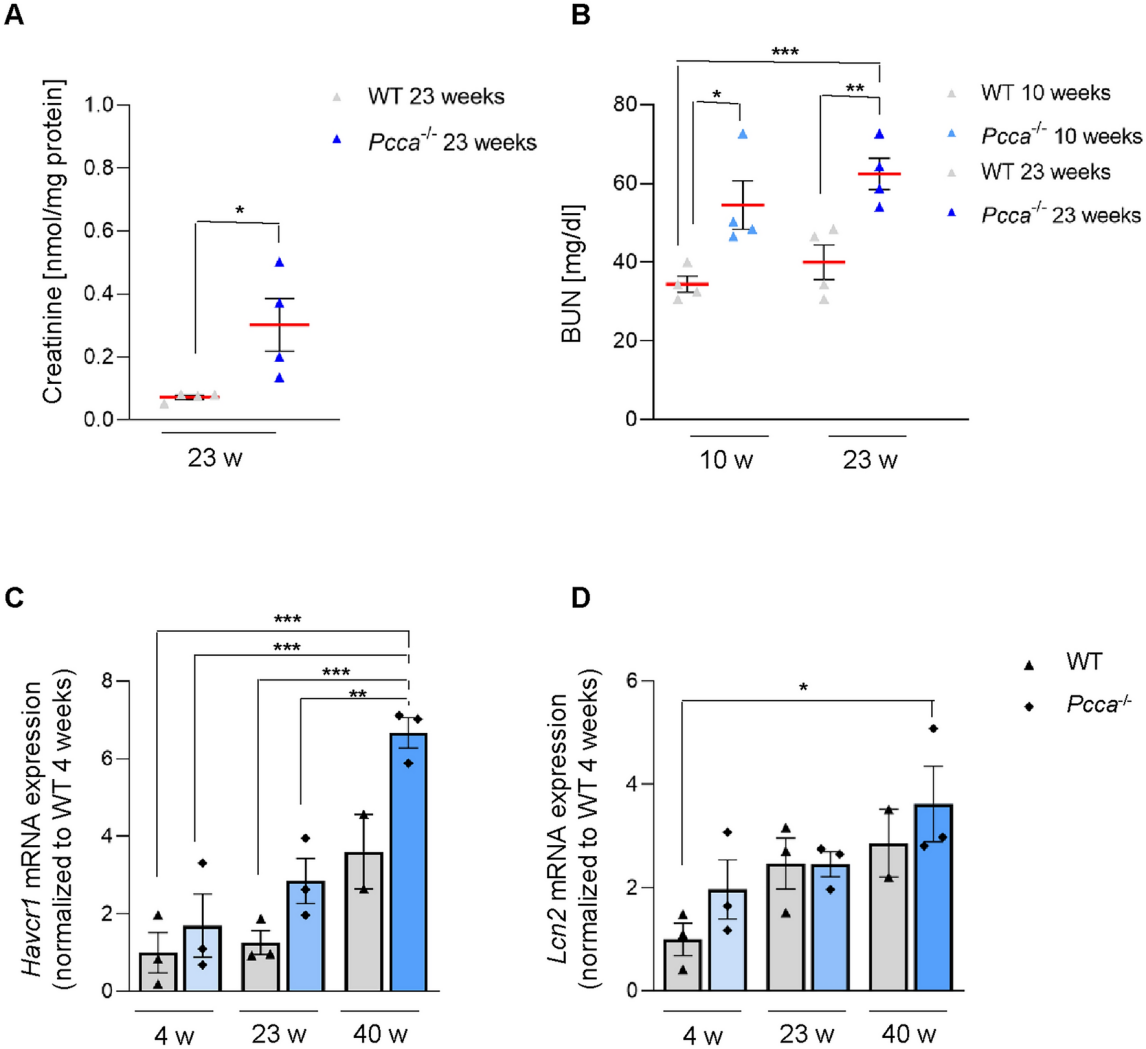
Kidney disease in *Pcca*^*-/-*^*(A138T)* mice. (**A**) LC–MS/MS quantification of creatinine concentrations in 23-week-old WT and *Pcca*^*-/-*^*(A138T)* mouse kidneys. Creatinine concentration in nmol/mg protein. n = 4 per group. (**B**) Blood urea nitrogen (BUN) concentration in the serum of 10- and 23-week-old WT and *Pcca*^*-/-*^*(A138T)* mice*.* BUN concentration in mg/dl. n = 4 per group. (**C**) Transcript levels of kidney injury molecule-1 (KIM-1, *Havcr1*) and lipocalin 2 (LCN2, *Lcn2*) obtained by RT-qPCR in kidney extracts of 4-, 23- and 40-week-old (w) WT and *Pcca*^*-/-*^*(A138T)* mice. n = 3 per group, n = 2 for WT 40-week-old mice.

We investigated serum levels, kidney lysates and sections of wild-type (WT) and *Pcca*^*-/-*^*(A138T)* mice at different time points. We performed our study on female mice, since male mice had a less pronounced renal phenotype (data not shown). LC–MS/MS revealed elevated creatinine levels in *Pcca*^*-/-*^*(A138T)* mice (2.5-fold) as compared to WT (Fig. 1A). The investigation of blood urea nitrogen (BUN) levels in the serum of *Pcca*^*-/-*^*(A138T)* mice showed an increase with time (Fig. 1B). RT-qPCR detected elevated transcription levels of Lipocalin2 (LCN2; *Lcn2*) and Kidney injury molecule-1 (KIM-1;*Havcr1)* indicating kidney damage (Fig. 1C,D) progressing over time.

**Fig. 2:**
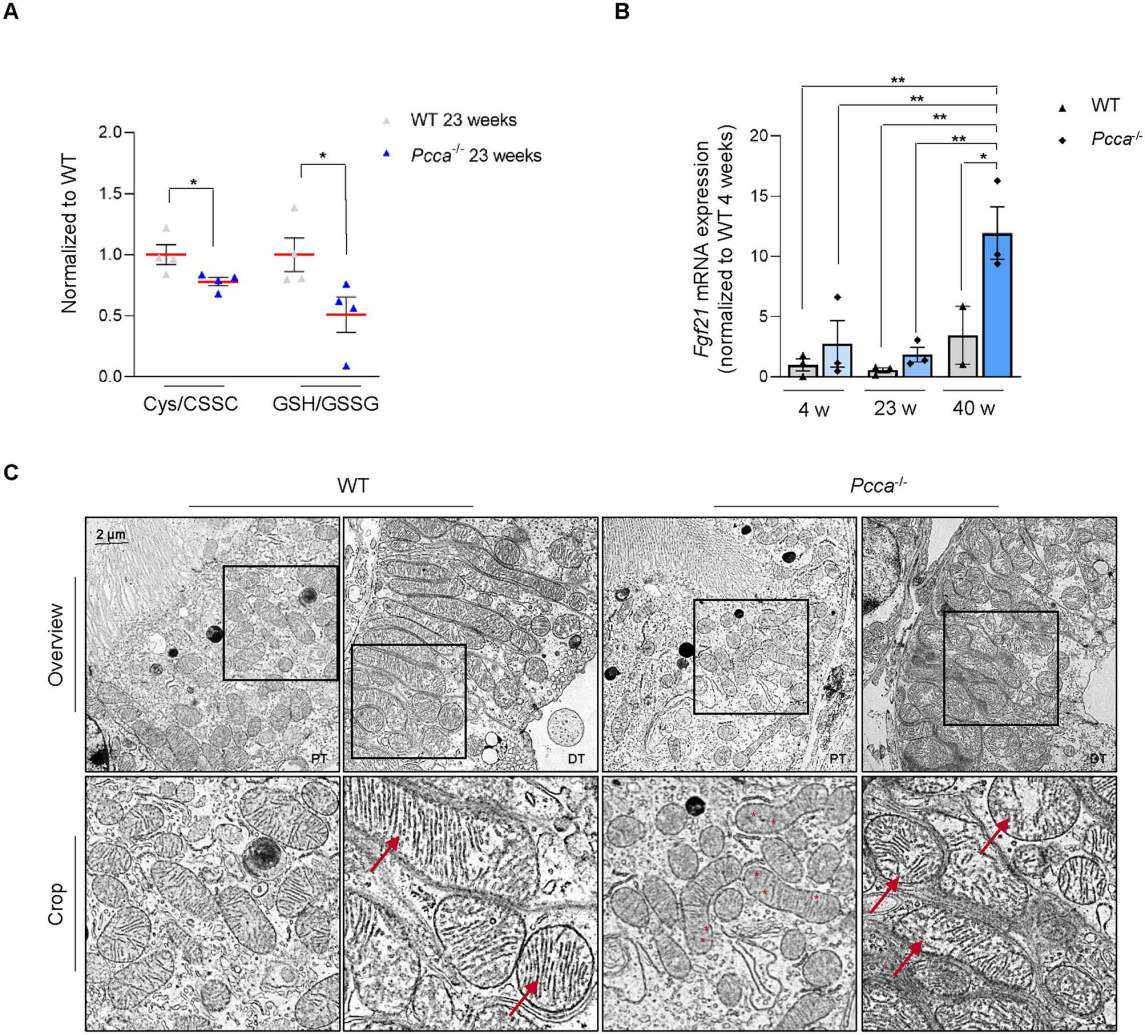
Altered mitochondrial morphology and function in *Pcca*^*-/-*^*(A138T)* mouse kidneys. (**A**) Quantitative LC–MS/MS analysis ratio of reduced (cysteine) to oxidized (CSSC) of cysteine and reduced (GSH) to oxidized (GSSG) levels of glutathione. Data normalized to WT. n = 4 per group. (**B**) Transcript levels of fibroblast growth factor 2 (FGF21, *Fgf21*) obtained by RT-qPCR in kidney extracts of 4-, 23- and 40-week-old (w) WT and *Pcca*^*-/-*^*(A138T)* mice. n = 3 per group, n = 2 for WT 40-week-old mice. (**C**) Representative transmission electron microscopy of proximal (PT) and distal tubular (DT) segments of 40-week-old WT and *Pcca*^*-/-*^*(A138T)* mouse kidneys (n = 2). Scale bar: 2 μm. n = 2 per group.

The ratios of reduced to oxidized glutathione and cysteine were reduced pointing to a compromised antioxidant system (Fig. 2A). Our study revealed elevated transcription levels of FGF21 (*Fgf21*) in *Pcca*^*-/-*^*(A138T)* mice which supports mitochondrial dysfunction (Fig. 2B). Electron microscopy revealed an extended, filamentous mitochondrial network with mainly preserved christae structure in WT mice. In contrast, the mitochondrial network was patchy in proximal and distal tubules of the *Pcca*^*-/-*^*(A138T)* model and showed disorganized, rarified networks suggesting structural alterations (Fig. 2C).

### Morphological assessment *Pcca*^*-/-*^*(A138T)* mouse kidneys

**Fig. 3:**
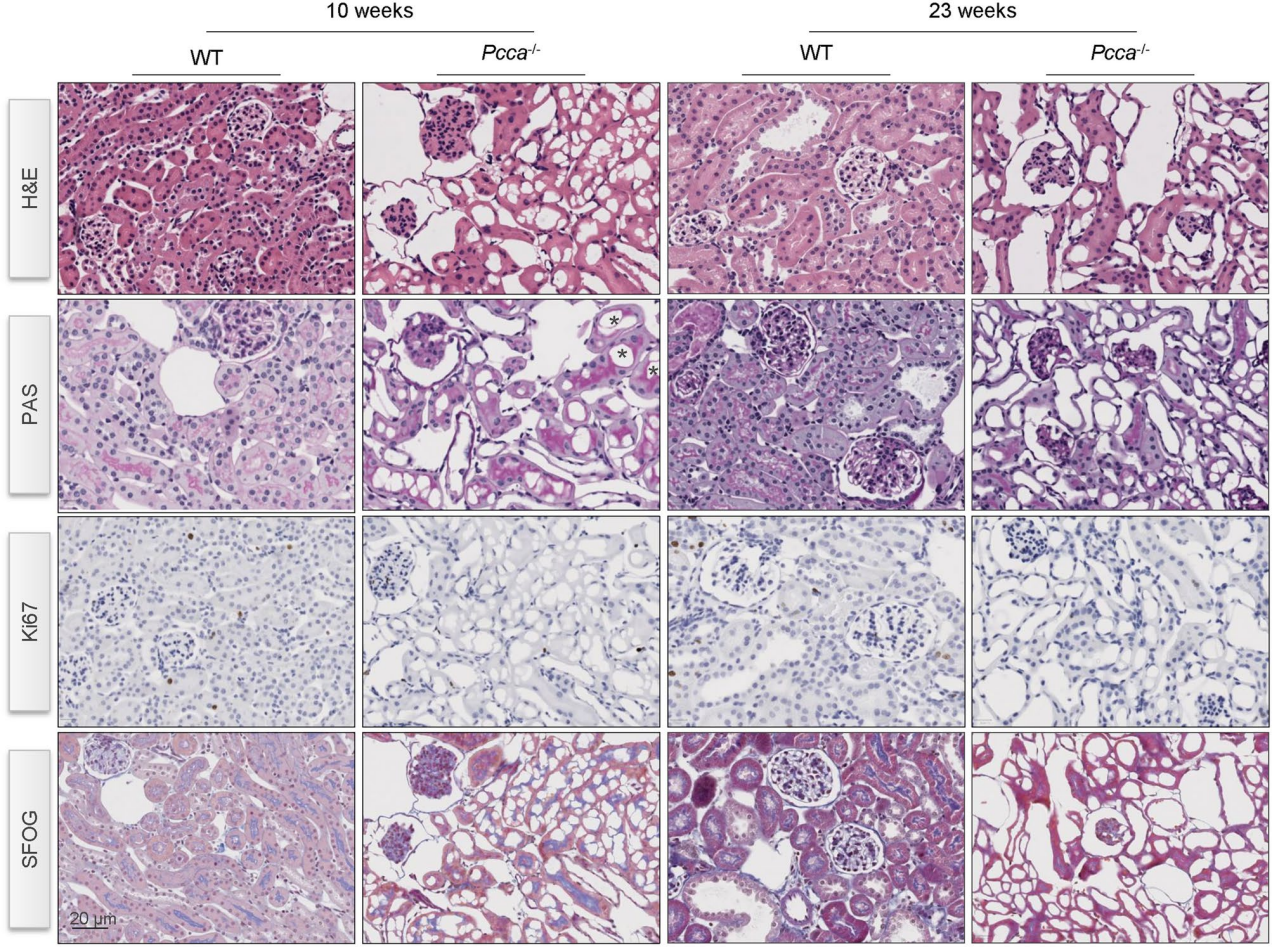
Partial flattening and focal tubular-cystic dilation in tubular cells of *Pcca*^*-/-*^*(A138T)* mice. Immunostaining in kidneys of WT and *Pcca*^*-/-*^*(A138T)* mice at 10 and 23 weeks of age addressing tissue structure and proliferation. H&E: hematoxylin and eosin staining*; PAS:* Periodic acid-Schiff staining; Ki67: Marker of proliferation Ki67; SFOG-staining: acid-fuchsin, orange-G, and aniline-blue staining. Representative images of n = 3 mice/group. Scale bar: 20 μm.

We performed immuno-histological studies on mouse kidneys at 10 and 23 weeks of age (Fig. 3). Morphological assessment of *Pcca*^*-/-*^*(A138T)* mouse kidneys showed a partial flattening of tubular epithelial cells and focal tubular-cystic dilation (Fig. 3). In some proximal tubular segments, we detected cytoplasmic vacuolization in *Pcca*^*-/-*^*(A138T)* mice. Fibrotic fibers or signs of inflammation were not identified (Fig. 3). Cellular proliferation was comparable in WT and *Pcca*^*-/-*^*(A138T)* mice. Employing SFOG staining (acid-fuchsin, orange-G, anilin-blue), no pronounced accumulation of extracellular matrix was detectable in WT and *Pcca*^*-/-*^*(A138T)* animals.

### Metabolic profiling of *Pcca*^*-/-*^*(A138T)* mouse kidneys

We used LC–MS/MS to measure disease-specific markers and tricarboxylic acid cycle (TCA) intermediates in WT and *Pcca*^*-/-*^*(A138T)* mouse kidneys. While lactate levels remained normal, methylcitrate concentrations were excessively elevated (34-fold, Fig. S1A). Propionate concentrations remained unchanged in *Pcca*^*-/-*^*(A138T)* mouse kidneys. MMA concentrations were in tendency higher (Fig. S1A).

Α-ketoglutarate is a TCA cycle intermediate involved in nitrogen metabolism and -via conversion to succinyl-CoA- in ATP production. We detected an increase in both α-ketoglutarate (1.8-fold) and succinate (twofold) concentrations (Fig. S1B) in *Pcca*^*-/-*^*(A138T)* mouse kidneys. Malate concentrations were elevated likewise (twofold, Fig. S1B). Both metabolites are linked via the α-ketoglutarate-malate carrier and provide the respiratory chain (RC) with NADH for ATP synthesis and NAD^+^ regeneration^15^. Malonate and itaconate are competitive inhibitors of succinate dehydrogenase (SDH), which is part of both the TCA cycle and the complex II^16^. Malonate and itaconate concentrations were elevated (1.7-fold and twofold (Fig. S1B)). Citrate is taken up from the circulation and largely contributes to energy generation via the renal TCA cycle^17^. We detected a tendency to higher citrate concentrations (1.7-fold) in the kidneys of *Pcca*^*-/-*^*(A138T)* mice (Fig. S1B).

We used acylcarnitine profiling as indirect read-out for fatty acid oxidation (FAO)^18^. Levels of free carnitine were comparable in WT and *Pcca*^*-/-*^*(A138T)* mice (Fig. S1C). Propionyl-carnitine concentrations (C3) were highly elevated in the PA mouse model (8.6-fold, Fig. S1C). Short-chain acylcarnitines were in tendency reduced, while medium- (MC) and long-chain (LC) were equally distributed (Fig. S1C). However, we observed a tendency towards accumulation of C8 and C10 acylcarnitines in *Pcca*^*-/-*^*(A138T)* mice (Fig. S1D).

The concentrations of the disease-associated amino acids glycine, isoleucine, methionine, valine and threonine remained unchanged (Fig. S2A) in *Pcca*^*-/-*^*(A138T)* mice. The ratio of alanine:lysine was markedly reduced, while the ratio of alanine:phenylalanine + tyrosine was comparable in WT and *Pcca*^*-/-*^*(A138T)* mice (Fig. S2B). Amino acid profiling revealed a steep increase of serine concentration (Fig. S2C). A reduction in the alanine:lysine ratio and elevated serine levels have been used as biomarkers for PA^19,20^.

### Regulation of mitochondrial mass and biogenesis in *Pcca*^*-/-*^*(A138T)* mouse kidneys

**Fig. 4:**
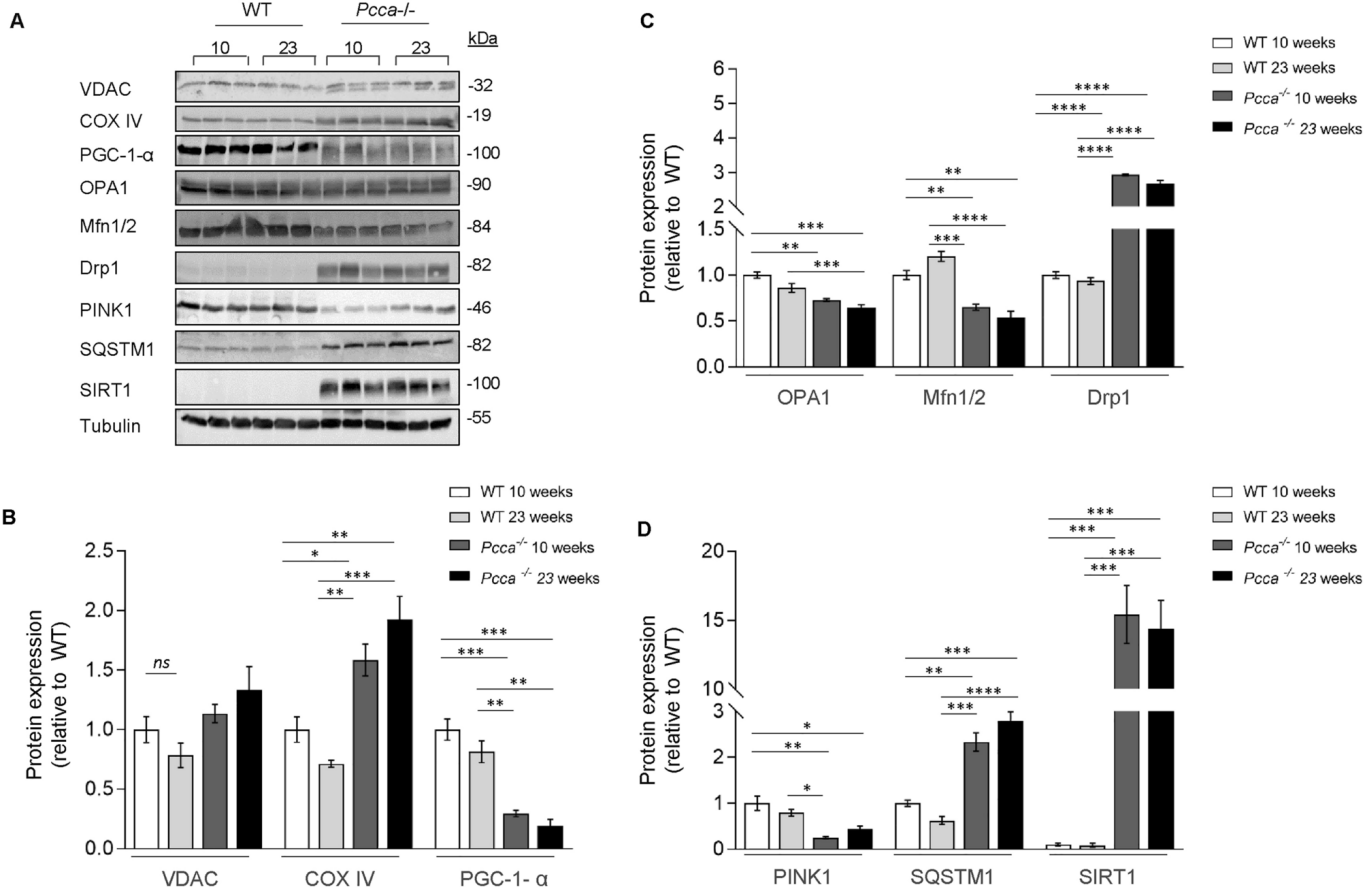
Protein expression profile of 10- and 23-week-old *Pcca*^*-/-*^*(A138T)* mice. (**A**) Immuno-blot analysis for mitochondrial homeostasis: voltage-dependent anion channel (VDAC1), cytochrome c oxidase IV (COX IV), peroxisome proliferator-activated receptor-gamma coactivator-1α (PGC-1-α); mitochondrial dynamics: optic atrophy-1 (OPA1), mitofusin1/2 (Mfn1/2), dynamin-related protein 1 (Drp1); mitochondrial quality control: sequestosome 1 (SQSTM1), PTEN-induced kinase 1 (PINK1), sirtuin1 (SIRT1). Tubulin was used as loading control. (**B**) Quantification of proteins for mitochondrial homeostasis. Data normalized to WT. n = 3 per group. (**C**) Quantification of proteins for mitochondrial dynamics. Data normalized to WT. n = 3 per group. (**D**) Quantification of proteins for mitochondrial quality control. Data normalized to WT. n = 3 per group.

Kidneys are highly energy dependent organs due to filtration and reabsorption processes. We performed immuno-blotting of the mitochondrial marker proteins voltage gated anion channel (VDAC1) and cytochrome c oxidase (COX IV) to evaluate potential mitochondrial adaption mechanisms. We observed an up-regulation of COX IV protein expression in the kidneys of 10- and 23-week old *Pcca*^*-/-*^*(A138T)* mice (1.6 and 1.8-fold) compared to WT littermates. Age did not affect VDAC1 and COX IV protein expression (Fig. 4A,B). Peroxisome proliferator-activated receptor-gamma coactivator (PGC-1-α) is the master regulator of mitochondrial biogenesis. PGC-1-α expression was markedly reduced (0.7-fold) in the kidneys of 10-week-old *Pcca*^*-/-*^*(A138T)* mice and slightly further reduced in 23-week-old littermates (Fig. 4A,B).The investigation of additional mitochondrial markers for different mitochondrial compartments by RT-qPCR (inner membrane: succinate dehydrogenase (*Sdh*), intermembrane space: cytochrome c (*Cycs*)) and matrix (pyruvate dehydrogenase subunit alpha (*Pdha1*); Fig. S3A-C)) showed comparable profiles of 4- and 23-week old animals, while 40-week-old WT mice showed a significant up-regulation of these mitochondrial transcripts.

### Alterations in mitochondrial dynamics and quality control

Mitochondrial networks constantly undergo coordinated reshaping to sustain ATP production and maintain cellular function, a process called “mitochondrial dynamics”^21^. Mitochondrial dynamics involves fission and fusion processes which are coordinated by the respective marker proteins dynamin related protein (Drp1, fission) and dynamin-related guanosine triphosphatase coded by the *OPA1* gene as well as mitofusin 1/2 (Mfn1/2, fusion)^22^. Immuno-blotting revealed a strong tendency to mitochondrial fission as evidenced by elevated Drp1 levels (threefold) in kidney lysates of *Pcca*^*-/-*^*(A138T)* animals, while the expression of the mitochondrial fusion proteins OPA1 (0.4-fold) and Mfn1/2 (0.5-fold) was markedly reduced (Fig. 4A,C) compared to WT mice. Again, aging did not aggravate the phenotype on protein level.

Having identified perturbations in mitochondrial dynamics and energy metabolism, we investigated the MQC system. Mitophagy is initiated by PTEN-induced kinase 1 (PINK1)^21^, and in times of mitochondrial stress and membrane depolarization, this protein is no longer cleaved but appears in large amounts on the surface priming mitochondria for degradation. SQSTM1 is a scaffold protein involved in activation of autophagy, a degradative cellular housekeeping pathway involved in cellular stress response^23^.

We detected markedly reduced levels (0.3- and 0.5-fold) for PINK1 in the kidneys of *Pcca*^*-/-*^*(A138T)* mice (Fig. 4A,D). General autophagy on the other hand was highly active as evidenced by up-regulation of SQSTM1 levels (2- and 2.7-fold, Fig. 4A,D) pointing at a need to compensate for defective mitochondrial clearance. NAD dependent deacetylase 1 (SIRT1) was up-regulated (up to 15-fold) in kidneys of *Pcca*^*-/-*^*(A138T)* mice (Fig. 4A,D).

## Discussion

The preservation of mitochondrial homeostasis and MQC are essential for energy dependent organs like the kidney^24^. Deficiency of mitochondrial PCC leads to ROS development in different tissues^11^ and accumulation of specific potentially toxic metabolites^1^. Recently, dysregulation of MQC and altered mitochondrial homeostasis were implicated in CKD development in OAs due to methylmalonyl-CoA mutase deficiency (MMA)^13,25^.

This study is the first providing insight into renal pathophysiology in a hypomorphic mouse model for PA. Our results suggest that renal involvement in OAs shares indeed common pathomechanisms. PCC deficiency leads to renal mitochondrial dysfunction and metabolic switches in energy metabolism. We detected unbalanced mitochondrial dynamics and MQC. The identified pathways offer therapeutic intervention potential and might be targeted to rescue kidney function in future studies.

*Pcca*^*-/-*^*(A138T)* mice develop CKD progressing with age, which is comparable to previously published data on renal cellular and mouse models for both PA^8^ and MMA^7,25^. Increased biomarkers of kidney injury (KIM1, LCN2) seem to be a shared feature in both OAs and thus might have potential as follow-up parameters in patients. Human renal biopsies in OAs are scarce -especially in PA. Tubulo-interstitial nephritis is characteristic in infantile and adult MMA patients^1^. However, Vernon reports focal dilatation, flattened epithelium and vacuolization in human tubular cells of an adult PA patient^5^. We detected partial flattening of tubular epithelial cells and focal tubular-cystic dilation in the kidneys of *Pcca*^*-/-*^*(A138T)* mice. Whether these histological differences might be one explanation for earlier onset and inevitable progression to end stage renal disease in MMA compared to PA needs further investigation.

Mitochondrial networks in proximal and distal tubular segments seemed less organized and patchier in *Pcca*^*-/-*^*(A138T)* mice highlighting the mitochondrion as target organelle in PA. Similar changes have been reported for a human renal tubular PA model^8^ and the few available patient biopsies^5^. Buchanan et al. detected that propionic acid, a key metabolite of PA, influences mitochondrial ultrastructure and dynamics in neuronal cells^26^. Studies in murine hepatic tissue and several renal cellular MMA models confirmed ultrastructural alterations in mitochondria^13^. Investigation of a mouse model for MMA revealed a slightly different pattern with abnormally shaped mitochondria and cristae rarefication primarily in proximal tubular cells^7^.

We detected alterations in the glutathione/thiol redox system with markedly reduced ratios of reduced to oxidized cysteine and glutathione indicating a need to counteract ROS. ROS production has been discussed as one possible pathomechanism promoting organ damage in both PA and MMA. Gallego Villar et al. reported elevated ROS levels in PA patients’ fibroblasts, which were positively correlated to the severity of the mutation and apoptotic rate^9^. Deep phenotyping of the *Pcca*^*-/-*^*(A138T)* mouse model revealed increased ROS levels in brain, heart and liver suggesting systemic ROS production and organ involvement in PA^10^. Isoleucine/valine load potentiated mitochondrial dysfunction in a renal cellular model for PA^8^. Anti-oxidant treatment ameliorated the phenotype in PA patients’ fibroblasts^27^ and different cellular and animal models for MMA^13,28^ highlighting the potential impact of ROS for organ damage in both OAs. FGF21 is a highly predictive disease biomarker for mitochondrial dysfunction in MMA independent of kidney function and correlating with disease severity^28^. We detected elevated expression levels of FGF21 progressing with age suggesting that metabolic stress response and disturbed mitochondrial function are a shared feature linking mitochondrial dysfunction to kidney damage in both OAs.

We next examined the impact of these alterations on the expression profiles of mitochondrial markers that label outer (VDAC) and inner membrane (*Sdh*, COX IV), intermembrane space (*Cycs*) and matrix (*Pdha1*). We detected increased protein levels of VDAC and COX IV as previously described for different renal PA^8^ and MMA models^13^. Transcript expression profiles of the other mitochondrial markers remained unchanged, however, an up-regulation in 40-week-old WT mice for *Sdh*, *Cycs* and *Pdha1* might suggest a relative lack in 40-week-old *Pcca*^*-/-*^*(A138T)* animals. Studies in human tissue of MMA and PA patients revealed reduced activities of respiratory chain complexes and TCA cycle enzymes^11^. We hypothesize that the up-regulation of mitochondrial markers could reflect a mechanism to compensate for deficient energy production via the RC in highly energy-depended renal cells but cannot comment on enzyme function.

The propionate pathway provides the TCA cycle with anaplerotic fuel via succinyl-CoA^12^ and energy derailment has been a matter of debate for both OAs. We observe marked elevations of α-ketoglutarate, citrate, itaconate, malate and succinate, which might be due to reduced enzymatic activity of TCA cycle enzymes. Inhibition of TCA cycle enzymes due to accumulation of disease-specific metabolites has been reported in tissues and patient cells in OAs^11,12,29^. The TCA cycle is tightly regulated and in constant feedback with OXPHOS. OXPHOS deficiency leads to NADH accumulation with negative impact on regulatory enzymes of the TCA cycle^30^. Both mechanisms could be involved in blocking substrate utilization in our PA model. Human renal cellular models for PA^8^ and MMA^25^ show a similar pattern. The elevation of succinate is striking, because depletion of anaplerotic substrates has been discussed as potential pathomechanism in OAs^31^. Due to reproducibility in different human renal cellular models^8,25^, our finding might reflect an organ-specific characteristic^32^.

Renal energy metabolism heavily relies on FAO fueling the TCA cycle. Impaired FAO is a feature of acute and chronic kidney disease and associated with renal fibrosis^33^. Clustering of medium-chain acylcarnitines revealed a trend towards higher levels in *Pcca*^*-/-*^*(A138T)* mice. We recently detected accumulation of medium and long-chain acylcarnitines in renal epithelial cells of PA and MMA patients^8,25^. The pattern was less pronounced in the PA models and might explain why fibrosis is not a predominant feature as compared to MMA. Forny et al. described accumulation of odd chain fatty acids in the plasma of a murine MMA model^34^ suggesting altered FAO as a shared feature in OAs. The underlying mechanisms await further investigation.

We next linked the metabolic profile to associated intracellular signaling pathways: PGC-1-α is the master regulator of MQC and controls gene expression of FAO and TCA cycle enzymes. Low levels of PGC-1-α might be responsible for the compromised performance of these players. Loss of PGC-1-α expression has been described in different renal models (e.g. diabetic kidney disease), associated with fibrosis^21^ and might be one possible explanation for CKD in PA. Multiple inductive and suppressing factors, which are active in renal injury and repair, control PGC-1-α expression and add complexity to this pathway^21^.

Mitochondria are highly dynamic networks constantly undergoing fission (Drp1) and fusion (OPA1, Mfn1/2) to adapt to environmental needs^21^. Mfn1/2 expression is positively correlated with PGC-1-α levels^35^, while we observed a strong tendency towards mitochondrial fission in our mouse model. Kidney research identified mitochondrial fission in CKD models of more “common” cause^36^ and might be a generalizable mechanism driving CKD of different origin.

Of note, high Drp1 levels suppress mitophagy and pharmacological Drp1 inhibition results in restauration of mitochondrial dynamics and preserves mitochondrial integrity by mitophagy initiation^36^.

Our investigation revealed a reduction of PINK1 levels in *Pcca*^*-/-*^*(A138T)* mice*.* We recently reported on defective mitophagy in MMA^13^ pointing towards a shared pathomechanism. Metabolic stress like protein and amino acid load further lowered already reduced PINK1 expression in a human renal MMA^25^ model, while human renal PA cells only showed reduced PINK1 levels in high protein exposure^8^. Over-expression of PINK1 in MUT deficient cells rescued mitophagy and supported lysosomal degradation^13^. Different models of diabetic nephropathy report on low expression of PINK1 accompanied by increased mitochondrial fragmentation^37,38^. Investigation of distinct disease entities like lung fibrosis^39^ and neurodegenerative disorders^40,41^ revealed a similar pattern. Reduction of ROS species rescued PINK1 expression and reversed CKD^38^. Interestingly, PGC-1-α (which is reduced in our model) exerts tissue specific effects and positively influences PINK1 expression in the kidney^42^. Activation of MQC protects against CKD of different origin^43^. We hypothesize that PINK1 deficiency (of primary or secondary course) is a rather common disease causing mechanism. The exact mode of action is unclear. The up-regulation of the autophagy marker SQSTM1^44^ might reflect a compensatory mechanism in a situation, where the removal of dysfunctional mitochondria is deficient.

Sirtuins are NAD^+^ dependent deacetylases and involved in regenerative processes such as antioxidative defence, anti- fibrotic effects and maintenance of mitochondrial function. SIRT1 is highly expressed in our model most likely to counter act the energetically and metabolically stressful conditions. SIRT1 positively regulates PGC-1-α activity through deacetylation. Strikingly, PGC-1-α is downregulated as previously observed for MMA^25^. We hypothesize that this could be due to several factors: Since deacetylation is a reversible process, PGC-1-α could be re-acylated by GCN5, an acetyltransferase with specific action on PGC-1-α^45^. PGC-1-α expression is negatively regulated by various other factors (e.g. GSK3-beta, Akt, S6K1, ubiquitine proteasome)^45,46^. GSK3-beta mediated phosphorylation and proteasomal degradation of PGC-1-α have been described in mice as a response against oxidative stress^39^. Epigenetic modifications block PGC-1-α at the transcription level or via demethylation. Transcription factors like HES1 have been inversely associated with PGC-1-α expression in human kidney and metabolic disease^45,46^. Finally, PGC-1-α expression relies on numerous environmental factors^47^. The complexity of possible influencing factors warrants further investigation, particularly since influencing PGC-1-α expression might be a promising therapeutic target.

Our study has several limitations. Due to the limited accessibility of the material, we examined molecular signatures in renal tissue only and the number of animals per group is rather small and the data need larger-scale confirmation. Only female mice developed a renal phenotype. Sex specific disease susceptibility has been reported in mouse models for MMA^7,34^ and been observed for both male and female mice. Different mouse strains and methods of genetic manipulation might be a reason. We cannot draw conclusions on the systemic effects of CKD on circulating metabolites in plasma and urine. The kidney extracts represent a blend of multiple cell types, and therefore, we cannot comment on individual contributions of different cell types to the renal phenotype. The relevance of the histopathological findings in mice for onset and progression of human CKD is unclear. All mentioned points warrant further investigation in future studies.

In sum, this study confirms disturbed mitochondrial homeostasis and MQC as potential CKD-driving mechanisms in PA and highlights the mitochondrion as target organelle for CKD development in OAs. The analysis sheds new light on pharmacologically targetable aims like the modulation of mitochondrial biogenesis using AMPK activators (e.g. metformin, AICAR), mitophagy/autophagy pathways (e,g, Tat-beclin1, resveratrol)^21,47^ or mitochondrial dynamics (e.g. mitochondrial division inhibitor 1). The evaluation of their therapeutic potential awaits further investigation.

## Material and methods

### Mice handling and isolation of kidneys

Mice kidneys used in this study were kindly provided by Prof. Dr. M. A. Barry (Mayo Clinic, Minnesota, US, generation of the model) and Prof. Dr. L.R. Desviat (Centro de Biología Molecular Severo Ochoa, Universidad Autónoma, Madrid, Spain, animal husbandry). The mouse model has been thoroughly characterized^48^.

All animal studies were approved by the Institutional Ethical Committee for Animal Experimentation (Universidad Autónoma de Madrid, reference CEI 963-A026 and CEI-134-2830) and by the Regional Environment Department (Comunidad de Madrid, reference PROEX 194/19). Animal housing and maintenance protocols followed the local authority guidelines. Mice were maintained on standard chow. All the experiments were carried out in a pathogen-free environment at the animal facility of the Centro de Biología Molecular Severo Ochoa, in accordance with the Spanish Law of Animal Protection. Food and water were available ad libitum, and mice were maintained in a temperature-controlled environment on a 12/12 h light–dark cycle. All mice used, wild-type (WT^+/+^) and hypomorphic *Pcca*^*-/-*^*(A138T)*, were females (4-, 10-, 23- and 40-week-old) in a C57BL/6 background. *Pcca*^*-/-*^*(A138T)* mice were obtained by crossing *Pcca*^-/-^ male with *Pcca*^+/-^ female mice to increase the number of *Pcca*^-/-^ animals. At the time/age stated for each experiment, mice were sacrificed in a carbon dioxide chamber and perfused with 50 mL of NaCl. Immediately after, kidneys were surgically excised. One kidney was snap-frozen in liquid nitrogen for RNA and protein extraction and stored at −80 °C, and the other was immersed in 4% PFA overnight and stored at 4 °C.

In order to obtain serum specimens, whole blood was collected by cardiac puncture and transferred to an Eppendorf tube. The blood samples were left for 1 h at 37 °C and at 4 °C overnight to facilitate coagulation, followed by two consecutive centrifugation steps (1200 × g, 5 min, and 4 °C) after which the respective supernatants were collected. The isolated serum fractions were stored at −80 °C.

Blood analysis was performed using the following kit according to the manufacturer’s instructions: Blood urea nitrogen (BUN): LT-UR; Labor&Technik, Eberhard Lehmann GmbH.

### RNA extraction

RNA was extracted from frozen kidneys of female WT and *Pcca*^*-/-*^*(A138T)* mice (4-week-, 23-week and 40-week-old animals) to study mRNA levels using real-time quantitative PCR. The total RNA extraction was carried out with the RNeasy Mini Kit (Qiagen, cat. no. 74104). For disruption of the tissue, a mortar and pestle were used. The tissue was frozen in liquid nitrogen and grinded to a fine powder under liquid nitrogen. The suspension was transferred to a new tube and mixed with 600 µl of lysis buffer. Homogenization war performed with QIAshredder columns (Qiagen, cat. no. 79654) by pipetting the lysate directly into a QIAshredder spin column and centrifuging for 2 min at full speed. After centrifugation (3 min at full speed), 70% ethanol was added to the flowthrough, and it was carefully mixed by pipetting. 700 μl of the sample were transferred to the RNeasy spin column and centrifuged for 15 s at 8000 × g. The column was washed with 700 μl RW1 buffer and centrifuged for 15 s at 8000 × g, followed by two subsequent washed with 500 μl RPE buffer and centrifugation at 8000 × g for 15 s and 2 min, respectively. The column was placed in a new collection tube and centrifuged for 2 min at 8000 × g. The RNA was eluted in 30 μl RNase-free water by centrifuging for 1 min at 8000 × g.

### Reverse transcription to cDNA

For reverse transcription of mRNA, the First Strand cDNA Synthesis Kit (Thermo Fisher Scientific, catalog number K1612) was used. If RNA yields were sufficient, 2 µg of RNA was utilized. The RNA, diluted in RNase-free water, was mixed with oligo(dT)_18_ primer and random hexamer primer (0.5 µl each) to a final volume of 11 µl. This mixture was incubated at 65 °C for 5 min and then placed on ice for at least 1 min.

A cDNA synthesis mix was then prepared, consisting of: 4 µl of 5 × Reaction Buffer, 1 µl of RiboLock RNase Inhibitor (20 U/µl), 2 µl of 10 mM dNTP mix, and 2 µl of M-MuLV Reverse Transcriptase (20 U/µl). A total of 9 µl of this cDNA synthesis mix was added to the RNA/primer mixture, gently mixed, briefly centrifuged, and incubated for 5 min at 25 °C, followed by 60 min at 37 °C. The reaction was terminated at 70 °C for 5 min and then chilled on ice.

### Real-time quantitative PCR

For real-time qPCR, the CFX96 Real-Time System (Bio-Rad Laboratories) with SYBR green labeling was used. The cDNA was diluted 1:10 in RNase-free water for the qPCR. A reaction mix was prepared containing 4 µl of SYBR Green iQ SuperMix (Bio-Rad Laboratories, catalog number 1708860), 0.5 µl of forward primer, 0.5 µl of reverse primer, and 5 µl of diluted cDNA. GAPDH served as the reference gene. Each well of a 96-well plate (Bio-Rad Laboratories, catalog number HSP9601) was filled with 10 µl of the reaction mix, and the plate was sealed with optical adhesive film. The real-time cycler was set up with an initial PCR activation step of 3 min at 95 °C, followed by 44 cycles of a 3-step program: 20 s at 95 °C for denaturation, 30 s at 58 °C for annealing, and 40 s at 72 °C for extension. Primer specificity for the detected product was confirmed by melt-curve analysis. The primers used were:

**Table:** 

Gene	Forward	Reverse
*Gapdh*	CCCATCACCATCTTCCAG	ATGACCTTGCCCACAGCC
*Cycs*	CCAAATCTCCACGGTCTGTTC	ATCAGGGTATCCTCTCCCCAG
*Fgf21*	CTGCTGGGGGTCTACCAAG	CTGCGCCTACCACTGTTCC
*Havcr1*	ACATATCGTGGAATCACAACGAC	ACAAGCAGAAGATGGGCATTG
*Lcn2*	TGGCCCTGAGTGTCATGTG	CTCTTGTAGCTCATAGATGGTGC
*Pdha1*	GAAATGTGACCTTCATCGGCT	TGATCCGCCTTTAGCTCCATC
*Sdh*	GGAACACTCCAAAAACAGACCT	CCACCACTGGGTATTGAGTAGAA

### Immuno-blotting

RIPA buffer was supplied with EDTA-free protease inhibitor cocktail (cOmplete, Roche, # 11697498001) and phosphatase inhibitor (PhosSTOP, Roche, # 0490683701). 100 mg renal tissue was grinded on ice with 800 µl of complete RIPA buffer for 1 min. The sample was centrifuged at 16,000 × g at 4 °C for 15 min and the supernatant collected. BCA assay (Pierce, Thermo Fisher Scientific) was used to determine the protein concentration. Samples were loaded using 100 µg protein/lane. SDS-PAGE was used for protein separation followed by blotting on a nitrocellulose membrane. Blocking was performed with 5% non-fat milk. The membranes were probed with the respective antibodies at 4 °C for 12 h, followed by incubation with the peroxidase-tagged secondary antibody (20 °C, 2 h) and developed using chemiluminescence (WBKLS0050, Millipore, Life technologies). γ- tubulin was used for protein normalization. Image J software (Image J 2.0.0-rc-68/1.52 g; Java 1.8.0_172 [64–bit]; https://imagej.net/ij/^49^) was used to quantify the density of each signal using for protein normalization.

### Antibodies used for immuno-blotting

Anti-PINK1 (1:400), ab23707, rabbit polyclonal (Abcam); anti- SQSTM1 (1:400), abMBL PM045, rabbit polyclonal (MBL International Corporation); anti-VDAC (1:1000), ab4661, rabbit monoclonal (Cell Signaling Technology); anti-DRP1 (1:200), rabbit monoclonal, ab8570 (Cell Signaling Technology); Anti-Lipocalin-2 (1:400), ab63929, rabbit polyclonal IgG antibody (Abcam), anti-OPA1 (1:500) rabbit monoclonal, ab80471 (Cell signaling Technology); anti-SIRT1 (1:500), rabbit polyclonal, ab2310 (Cell Signaling Technology), anti-COX IV (1:500), ab14744, mouse monoclonal (Abcam); anti- PGC1-α (1:500), ab19838, rabbit polyclonal (Abcam); anti-Mitofusin1/2 (1:1000), ab57602, mouse monoclonal (Abcam); anti-gamma-Tubulin (1:1000), ab T5326, mouse monoclonal (Sigma).

### Quantitative profiling of metabolites by liquid chromatography and mass spectrometry (LC–MS/MS)

Murine renal tissue biopsies were thawed and homogenised with ice-cold PBS supplemented with 1% protease inhibitor cocktail (Sigma Nr. P8340-5 mL), using a cordless pestle motor and disposable pellet mixers (VWR Nr. 47747-366). Buffer volume was adjusted such to reach a target of 0.1 to 0.3 mg wet-tissue/mL lysis buffer. Whole tissue homogenates were aliquoted for downstream measurement of total protein concentration, flash-frozen and stored at −80 °C. All metabolites as well as creatinine and the redox thiol pools were determined according to a previously published procedure^50^. Lactate, TCA and glycolysis intermediates and other organic acids, and folates were determined as described in previous work^51^. Amino acids and neurotransmitters were determined using a previously described protocol^52,53^. A commercially available standardized amino acid mixture was utilized to generate a calibration curve for amino acids (Amino acid standards, physiological, Sigma, Nr. A9906-10 mL). Calibration curves for all other metabolites were prepared from individual stock solutions prepared in house. Quantitation accuracy was examined by monitoring homocysteine and methylmalonic acid concentrations in an external quality control, namely, the Control Special Assays in Serum, European Research Network for the evaluation and improvement of screening, diagnosis, and treatment of Inherited disorders of Metabolism (ERNDIM) IQCS, SAS-02.1 and SAS-02.2 from MCA Laboratories, Winterswijk, Netherlands. For all other metabolites, quantitation trueness was tested by examining metabolite concentrations in plasma from a previously validated sample isolated from a healthy control individual with respect to standard reference ranges, using the same calibration curves and LC–MS/MS running conditions. Quantification of metabolites was carried out with Analyst 1.7.2 software, 2022 AB Sciex (https://sciex.com/products/software/analyst-software).

### Transmission electron microscopy

Mice were sacrificed in a carbon dioxide chamber and perfused through the left ventricle with with 50 mL of NaCl and then with a solution of 4% formaldehyde and 2% glutaraldehyde in 0.1 M phosphate buffer pH 7.4. Kidneys were postfixed in the same solution for 2 h at room temperature and overnight at 4 °C. Upper kidney pole was cut into transverse slabs that were postfixed in 1% osmium tetroxide in 0.1 M cacodylate buffer, dehydrated in ethanol and embedded in Epon-Araldite. Serial ultrathin sections of transverse section of kidneys were collected on pioloform-coated, single-hole grids, and stained with uranyl acetate and lead citrate. The sections were analysed with a JEM-1010 transmission electron microscope (Jeol, Japan) equipped with a side-mounted CCD camera Mega View III from Olympus Soft Imaging System GmBH (Muenster, Germany, https://www.olympus-lifescience.com). Mitochondria of 40-week-old mice were sampled randomly in the distal and proximal tubules of the kidney at a magnification of 3000X.

### Statistical analysis

Data represent mean ± SEM. Data were first tested with an F test to compare variances. More than 2 groups were compared with ANOVA using Tukey’s correction; in the cases where variances among the groups were significantly different, Browne-Forsythe and Welch ANOVA test with Dunnett’s test was used. GraphPad Prism 9 software (https://www.graphpad.com) was used for the analyses. *P < 0.05, **P < 0.01, ***P < 0.001, and ****P < 0.0001: *ns*: not significant. Quantitative analysis (metabolite concentrations in tissue nmol/mg protein) are summarized in the supplementary. All values are displayed as compared to WT.

## Supplementary Information

Supplementary Information 1.

Supplementary Information 2.

Supplementary Information 3.

Supplementary Information 4.

## Data Availability

The datasets reported are available upon reasonable request to the corresponding author.
