# Supplementary material for: Renal phenotyping in a hypomorphic murine model of propionic aciduria reveals common pathomechanisms in organic acidurias
Source: Sci Rep. 2024 Dec 16;14:30478. doi: 10.1038/s41598-024-79572-z (PMC11649940; doi:10.1038/s41598-024-79572-z)
Supplement: Supplementary file 1 — Supplementary Information 1. [file 41598_2024_79572_MOESM1_ESM.pdf]

Supplementary material

Supplementary table 1:

(A)

Group	Creatinine (nmol/mg protein)
WT	0,080
WT	0,081
WT	0,076
WT	0,052
Pcca$^{-/-}$	0,135
Pcca$^{-/-}$	0,200
Pcca$^{-/-}$	0,502
Pcca$^{-/-}$	0,372

(B)

Age	Group	BUN (mg/dl)	Age	Group	BUN (mg/dl)
10 weeks	WT	30,650	23 weeks	WT	48,411
	WT	34,390		WT	46,542
	WT	32,520		WT	30,654
	WT	40,000		WT	34,393
	Pcca-/-	48,410		*Pcca*-/-	72,710
	Pcca-/-	72,710		*Pcca*-/-	64,299
	Pcca-/-	46,540		*Pcca*-/-	58,692
	Pcca-/-	50,280		*Pcca*-/-	54,019

Supplementary table 1: **(A)** Absolute concentrations of creatinine measured in crude kidney lysates of 23 week old WT and *Pcca*$^{-/-}$(A138T) mice. Values expressed as nmol/mg protein. N=4. **(B)** Absolute concentrations of blood urea nitrogen (BUN) measured in the serum of 10 and 23 week old WT and *Pcca*$^{-/-}$(A138T) mice. Values expressed as mg/dl.

Supplementary table 2:

	Aminothiols (nmol/mg protein)			
Group	Free Cysteine	CSSC	Free Glutathione	GSSG
WT	16,348	4,666	0,073	0,004
WT	10,335	3,735	0,037	0,003
WT	13,695	4,897	0,056	0,006
WT	16,254	6,758	0,047	0,005
Pcca$^{-/-}$	10,134	5,195	0,064	0,008
Pcca$^{-/-}$	7,528	3,142	0,030	0,004
Pcca$^{-/-}$	10,176	4,358	0,050	0,005
Pcca$^{-/-}$	13,369	5,912	0,003	0,002

Supplementary table 2: Absolute concentrations of reduced (cysteine) and oxidized cysteine (CSSC) and reduced (glutathione) and oxidized (GSSG) glutathione measured in crude kidney lysates of 23 week old WT and *Pcca*$^{-/-}$(A138T) mice. Values expressed as nmol/mg protein.

Supplementary table 3:

Group	Alanine	Arginine	Asparagine	Aspartic Acid	GABA	Glutamine	Glutamic Acid	Glycine	Isoleucine/Leucine
	Amino acids in nmol/mg protein								
WT	1,695	1,623	288,783	1,301	0,240	1,396	9,785	429,594	2,220
WT	1,156	0,954	156,434	1,029	0,156	1,024	7,328	283,741	1,372
WT	1,262	1,107	181,536	1,095	0,254	1,250	8,201	390,541	1,696
WT	1,798	1,550	192,138	1,559	0,366	1,555	10,336	472,615	2,288
$Pcca^{-/-}$	1,184	1,064	149,025	2,861	0,150	0,801	10,645	312,527	1,524
$Pcca^{-/-}$	1,402	1,198	133,272	1,893	0,165	1,310	7,182	311,480	1,844
$Pcca^{-/-}$	1,376	1,357	135,680	3,066	0,129	1,267	8,448	420,480	1,690
$Pcca^{-/-}$	1,299	1,284	173,946	2,697	0,140	1,246	8,887	336,498	1,588

Group	Histidine	Lysine	Methionine	Phenylalanine	Proline	Serine	Threonine	Tryptophane	Tyrosine	Valine
	Amino acids in nmol/mg protein									
WT	0,476	1,820	1,527	1,796	1,331	431,384	1,331	6,921	0,503	2,339
WT	0,275	1,019	0,545	1,174	0,775	396,956	0,794	6,624	0,300	1,433
WT	0,336	1,318	1,027	1,477	1,119	265,934	0,932	4,618	0,364	1,736
WT	0,451	1,612	1,290	1,966	1,590	274,293	1,409	6,670	0,504	2,301
$Pcca^{-/-}$	0,376	1,431	1,022	1,294	0,886	676,148	0,886	11,581	0,380	1,541
$Pcca^{-/-}$	0,362	1,559	1,133	1,648	1,018	642,601	0,991	5,031	0,419	1,859
$Pcca^{-/-}$	0,365	1,638	1,178	1,491	0,973	495,360	0,960	8,384	0,403	1,709
$Pcca^{-/-}$	0,362	1,686	1,056	1,474	0,912	493,733	0,912	7,596	0,388	1,664

Supplementary table 3: Absolute concentrations of amino acids measured in crude kidney lysates of 23 week old WT and $Pcca^{-/-}$(A138T) mice. N=4. Values expressed as nmol/mg protein.

Supplementary table 4:

(A)

Group	alpha-ketoglutarate	Citrate	Ethylmalonate	Itaconate	Malate	Malonate	Succinate
	TCA metabolites in nmol/mg protein						
WT	1,998	0,132	0,047	0,097	4,140	1,338	0,324
WT	2,321	0,038	0,059	0,039	4,648	1,718	0,529
WT	2,231	0,047	0,027	0,042	3,368	1,461	0,413
WT	1,877	0,059	0,023	0,087	3,344	1,281	0,439
$Pcca^{-/-}$	3,916	0,091	0,048	0,114	7,611	2,575	0,761
$Pcca^{-/-}$	3,021	0,074	0,140	0,084	4,666	1,984	0,749
$Pcca^{-/-}$	4,002	0,130	0,039	0,170	9,963	2,657	1,005
$Pcca^{-/-}$	3,752	0,108	0,100	0,120	7,444	2,438	0,745

(B)

Group	Lactate	MMA	Methylcitrate	Propionate
	Disease specific metabolites in nmol/mg protein			
WT	60,114	0,016	0,010	0,032
WT	71,599	0,019	0,012	0,034
WT	54,963	0,014	0,007	0,031
WT	60,071	0,009	0,004	0,058
$Pcca^{-/-}$	45,704	0,027	0,176	0,035
$Pcca^{-/-}$	64,640	0,017	0,267	0,037
$Pcca^{-/-}$	80,303	0,021	0,403	0,073
$Pcca^{-/-}$	75,959	0,030	0,251	0,039

Supplementary table 4: **(A)** Absolute concentrations of tricarboxylic citric acid cycle metabolites in crude kidney lysates of 23 week old WT and $Pcca^{-/-}$(A138T) mice. **(B)** Absolute concentrations of disease specific metabolites in crude kidney lysates of 23 week old WT and $Pcca^{-/-}$(A138T) mice. N=4. Values expressed as nmol/mg protein.

Supplementary table 5:

	Acylcarnitines in nmol/mg protein							
**Metabolite**	**WT**	**WT**	**WT**	**WT**	***Pcca*** $^{-/-}$	***Pcca*** $^{-/-}$	***Pcca*** $^{-/-}$	***Pcca*** $^{-/-}$
**C0**	3,491	2,042	4,422	3,314	3,279	2,972	3,514	3,677
**C2**	1,262	0,938	1,396	1,308	1,018	0,899	1,024	0,975
**C3**	0,014	0,010	0,014	0,011	0,052	0,083	0,079	0,203
**C4**	0,011	0,007	0,007	0,007	0,004	0,006	0,005	0,007
**C4OH**	0,019	0,016	0,027	0,019	0,012	0,013	0,016	0,017
**C4DC**	0,017	0,017	0,020	0,012	0,007	0,012	0,013	0,011
**C5 + C4:1OH**	0,005	0,005	0,006	0,006	0,007	0,010	0,008	0,011
**C5:1**	0,016	0,025	0,026	0,030	0,004	0,030	0,027	0,021
**C5OH**	0,026	0,021	0,023	0,023	0,023	0,029	0,023	0,026
**C6**	0,012	0,026	0,024	0,017	0,004	0,029	0,032	0,017
**C6:1**	0,011	0,006	0,007	0,006	0,004	0,008	0,006	0,006
**C6OH**	0,018	0,015	0,019	0,021	0,008	0,017	0,024	0,017
**C6DC**	0,025	0,019	0,023	0,021	0,008	0,031	0,020	0,019
**C8**	0,012	0,019	0,019	0,014	0,007	0,021	0,029	0,011
**C10**	0,037	0,032	0,040	0,031	0,048	0,055	0,031	0,057
**C10:1**	0,024	0,019	0,026	0,027	0,004	0,014	0,023	0,017
**C10:2**	0,014	0,020	0,018	0,016	0,003	0,017	0,021	0,011
**C12**	0,014	0,014	0,014	0,018	0,003	0,012	0,020	0,010
**C12OH**	0,024	0,026	0,033	0,036	0,005	0,034	0,036	0,024
**C14**	0,008	0,007	0,005	0,007	0,004	0,008	0,007	0,011
**C14:1**	0,007	0,006	0,009	0,007	0,003	0,007	0,008	0,007
**C14:2**	0,007	0,007	0,009	0,008	0,004	0,008	0,011	0,013
**C14OH**	0,014	0,017	0,021	0,022	0,003	0,026	0,019	0,020
**C16**	0,015	0,011	0,014	0,015	0,008	0,010	0,016	0,009
**C16OH**	0,010	0,008	0,016	0,017	0,012	0,039	0,014	0,013
**C18**	0,011	0,013	0,014	0,014	0,005	0,015	0,010	0,009
**C18:1**	0,010	0,012	0,017	0,015	0,006	0,010	0,022	0,007
**C18:2**	0,011	0,012	0,013	0,012	0,005	0,009	0,016	0,007
**C18OH**	0,009	0,008	0,013	0,013	0,003	0,012	0,013	0,011
**C18:1OH**	0,011	0,008	0,012	0,014	0,003	0,013	0,013	0,011
**C18:2OH**	0,013	0,012	0,014	0,011	0,005	0,017	0,018	0,012

Supplementary table 5: Absolute concentrations of acylcarnitines in crude kidney lysates of 23 week old WT and *Pcca* $^{-/-}$(A138T) mice. Values expressed as nmol/mg protein.

Supplementary figure 6:

(A)

Gene	Genotype	Ct value 4 weeks	23 weeks	40 weeks
Gapdh	WT	15,56	15,99	18,74
Gapdh	WT	15,14	15,99	18,92
Fgf21	WT	28,63	34,54	32,47
Fgf21	WT	28,91	30,35	32,33
Lcn2	WT	25,95	24,81	28,23
Lcn2	WT	25,64	25,00	28,31
Havcr1	WT	27,94	28,78	29,86
Havcr1	WT	28,00	28,14	29,71
Sdh	WT	22,35	22,04	21,32
Sdh	WT	22,34	22,21	21,35
Cycs	WT	17,66	18,58	18,21
Cycs	WT	17,80	18,84	18,17
Pdha1	WT	23,41	23,37	23,02
Pdha1	WT	23,04	23,32	22,66
Gapdh	WT	16,52	14,98	17,49
Gapdh	WT	15,84	14,73	17,16
Fgf21	WT	28,63	29,43	28,46
Fgf21	WT	29,31	28,53	28,36
Lcn2	WT	26,02	23,87	26
Lcn2	WT	26,36	24,12	26,18
Havcr1	WT	27,47	26,24	27,62
Havcr1	WT	27,64	26,37	27,36
Sdh	WT	23,51	21,90	20,51
Sdh	WT	23,06	22,18	20,74
Cycs	WT	18,33	17,67	17,75
Cycs	WT	18,55	17,85	17,83
Pdha1	WT	23,82	22,92	22,15
Pdha1	WT	24	22,87	21,93
Gapdh	WT	15,32	15,29	-
Gapdh	WT	14,93	15,37	-
Fgf21	WT	33,33	29,37	-
Fgf21	WT	33,35	29,16	-
Lcn2	WT	26,88	25,26	-
Lcn2	WT	27,04	25,35	-
Havcr1	WT	29,82	27,88	-
Havcr1	WT	29,91	27,58	-
Sdh	WT	19,64	20,96	-
Sdh	WT	19,66	20,91	-
Cycs	WT	17,03	17,13	-
Cycs	WT	17,10	17,31	-
Pdha1	WT	21,12	22,34	-
Pdha1	WT	21,42	22,38	-

(B)

Gene	Genotype	Ct value 4 weeks	23 weeks	40 weeks
Gapdh	*Pcca*$^{-/-}$	16,03	16,09	16,96
Gapdh	*Pcca*$^{-/-}$	15,92	16,07	16,69
Fgf21	*Pcca*$^{-/-}$	29,45	29,21	27,34
Fgf21	*Pcca*$^{-/-}$	29,41	29,31	27,12
Lcn2	*Pcca*$^{-/-}$	26,38	25,34	25,95
Lcn2	*Pcca*$^{-/-}$	26,26	25,06	25,71
Havcr1	*Pcca*$^{-/-}$	28,32	27,07	26,54
Havcr1	*Pcca*$^{-/-}$	28,09	27,02	26,71
Sdh	*Pcca*$^{-/-}$	21,79	23,12	23,12
Sdh	*Pcca*$^{-/-}$	21,67	22,94	23,93
Cycs	*Pcca*$^{-/-}$	18,02	18,48	20,28
Cycs	*Pcca*$^{-/-}$	18,00	18,50	20,45
Pdha1	*Pcca*$^{-/-}$	23,23	24,11	24,61
Pdha1	*Pcca*$^{-/-}$	23,03	23,92	24,38
Gapdh	*Pcca*$^{-/-}$	15,44	16,11	18,95
Gapdh	*Pcca*$^{-/-}$	15,37	16,07	18,68
Fgf21	*Pcca*$^{-/-}$	27,09	29,97	29,07
Fgf21	*Pcca*$^{-/-}$	25,54	29,17	29,14
Lcn2	*Pcca*$^{-/-}$	24,34	25,74	27,86
Lcn2	*Pcca*$^{-/-}$	24,39	25,65	27,95
Havcr1	*Pcca*$^{-/-}$	26,02	27,40	28,29
Havcr1	*Pcca*$^{-/-}$	26,05	27,54	28,39
Sdh	*Pcca*$^{-/-}$	22,29	21,56	23,71
Sdh	*Pcca*$^{-/-}$	22,17	21,45	23,74
Cycs	*Pcca*$^{-/-}$	18,85	17,91	20,46
Cycs	*Pcca*$^{-/-}$	18,40	17,98	20,73
Pdha1	*Pcca*$^{-/-}$	23,36	22,73	25,14
Pdha1	*Pcca*$^{-/-}$	23,09	22,74	25,27
Gapdh	*Pcca*$^{-/-}$	16,01	18,30	17,03
Gapdh	*Pcca*$^{-/-}$	15,74	18,22	16,92
Fgf21	*Pcca*$^{-/-}$	30,04	30,51	26,15
Fgf21	*Pcca*$^{-/-}$	31,03	30,06	27,02
Lcn2	*Pcca*$^{-/-}$	25,80	27,48	25,29
Lcn2	*Pcca*$^{-/-}$	25,67	27,39	25,13
Havcr1	*Pcca*$^{-/-}$	28,72	28,69	26,39
Havcr1	*Pcca*$^{-/-}$	28,85	28,58	26,65
Sdh	*Pcca*$^{-/-}$	20,59	23,09	25,68
Sdh	*Pcca*$^{-/-}$	20,81	23,08	25,56
Cycs	*Pcca*$^{-/-}$	17,07	20,11	20,77
Cycs	*Pcca*$^{-/-}$	17,09	20,34	20,7
Pdha1	*Pcca*$^{-/-}$	21,92	24,62	25,13
Pdha1	*Pcca*$^{-/-}$	22,22	24,59	25,11

Supplementary figure 6: Ct values of the investigated genes in 4, 23 and 40 week old WT **(A)** and *Pcca*$^{-/-}$(A138T, **(B)**) mice. Glyceraldehyde-3-phosphate dehydrogenase (*Gapdh*), Fibroblast growth factor 21 (*Fgf21*), Lipocalin2 (*Lcn2*), Kidney injury molecule 1 (*Havcr1*), Succinate dehydrogenase (*Sdh*), Cytochrome c (*Cycs*), Pyruvate dehydrogenase (*Pdha1*).
