# Supplementary figures and images for: Renal phenotyping in a hypomorphic murine model of propionic aciduria reveals common pathomechanisms in organic acidurias
Source: Sci Rep. 2024 Dec 16;14:30478. doi: 10.1038/s41598-024-79572-z (PMC11649940; doi:10.1038/s41598-024-79572-z)

Supplementary figure 1

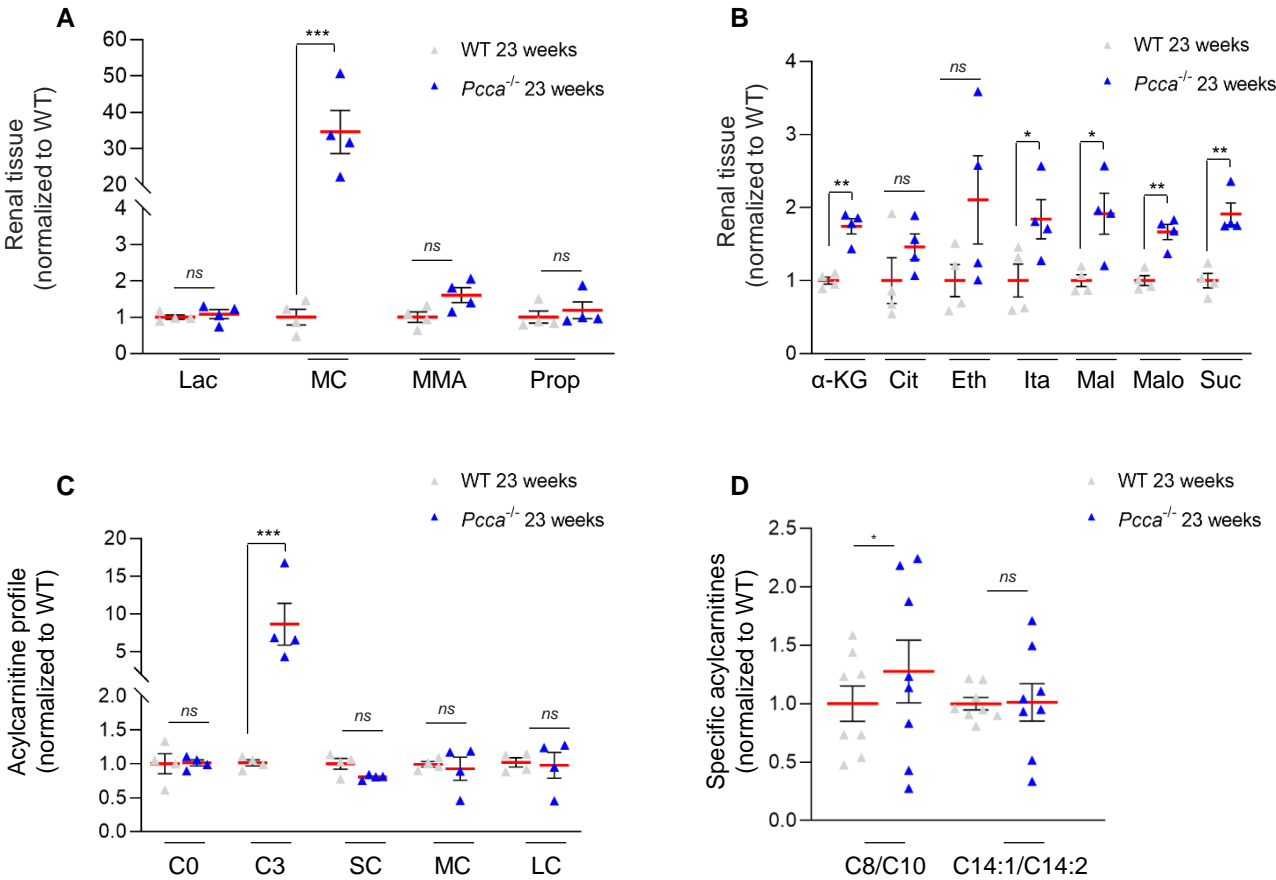

Supplementary figure 2

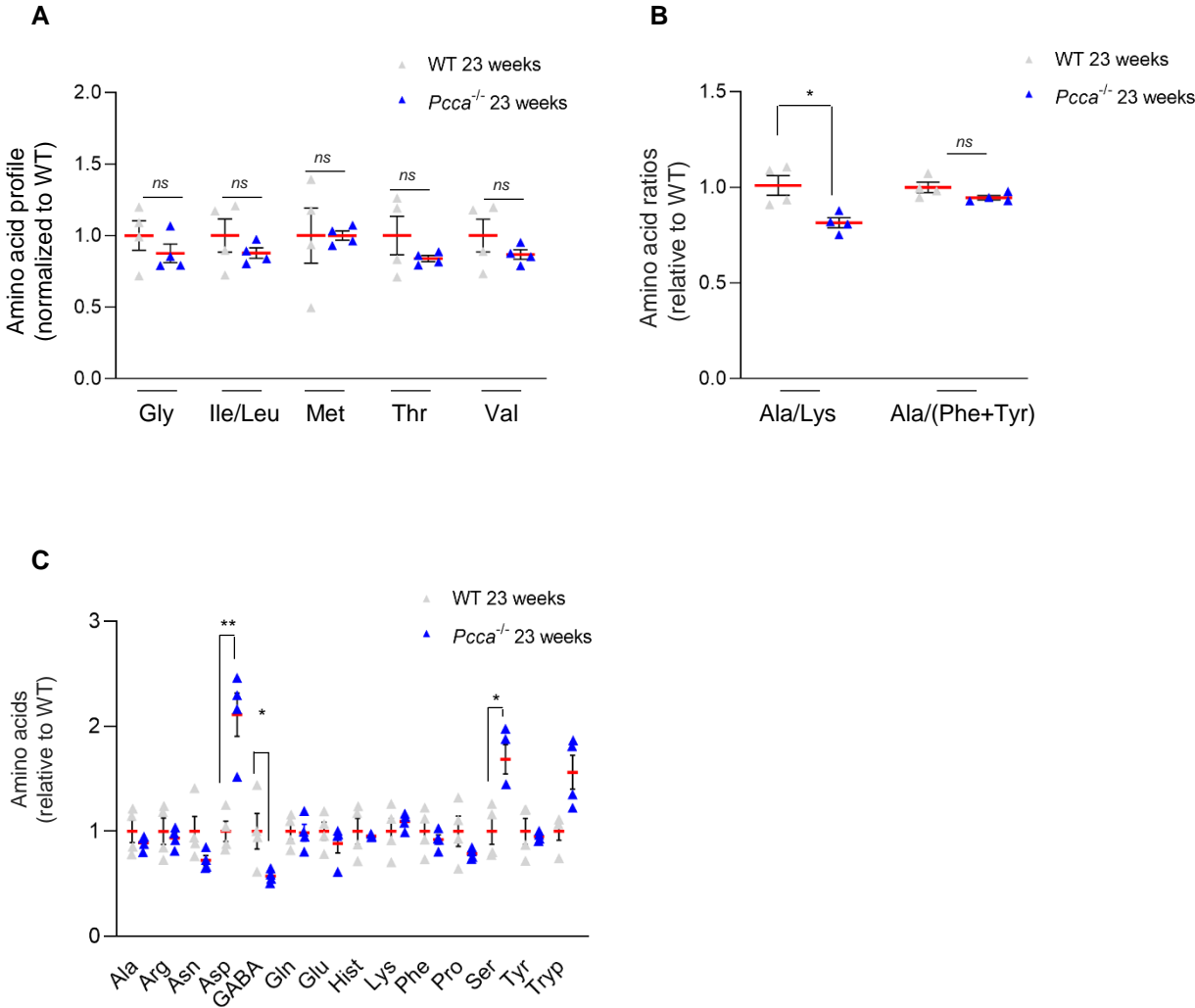

Supplementary figure 3

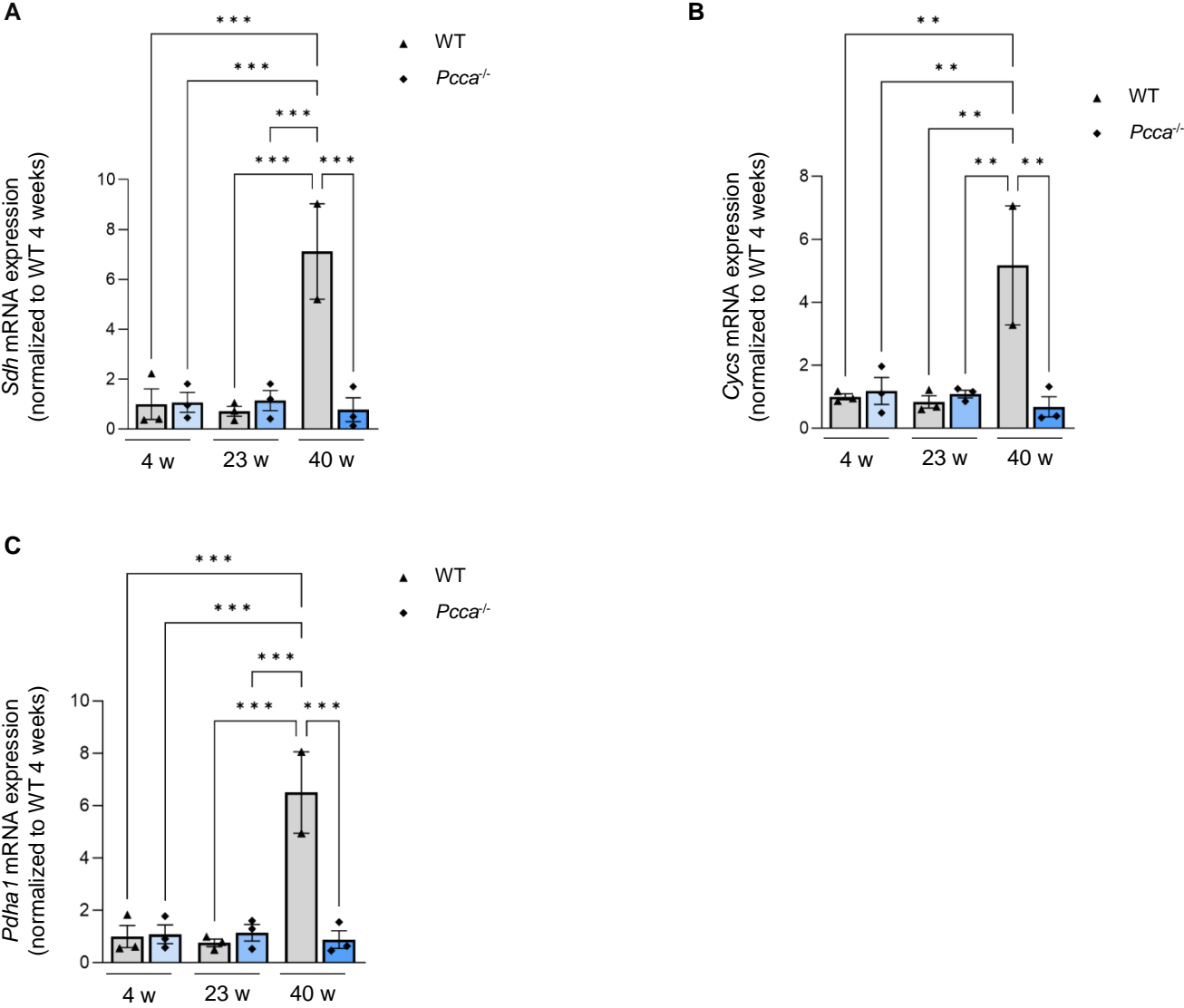

Supplement: Supplementary file 2 — Supplementary Information 2. [file 41598_2024_79572_MOESM2_ESM.pdf]
