# Supplementary material for: Renal phenotyping in a hypomorphic murine model of propionic aciduria reveals common pathomechanisms in organic acidurias
Source: Sci Rep. 2024 Dec 16;14:30478. doi: 10.1038/s41598-024-79572-z (PMC11649940; doi:10.1038/s41598-024-79572-z)
Supplement: Supplementary file 3 — Supplementary Information 3. [file 41598_2024_79572_MOESM3_ESM.pdf]

Supplementary material

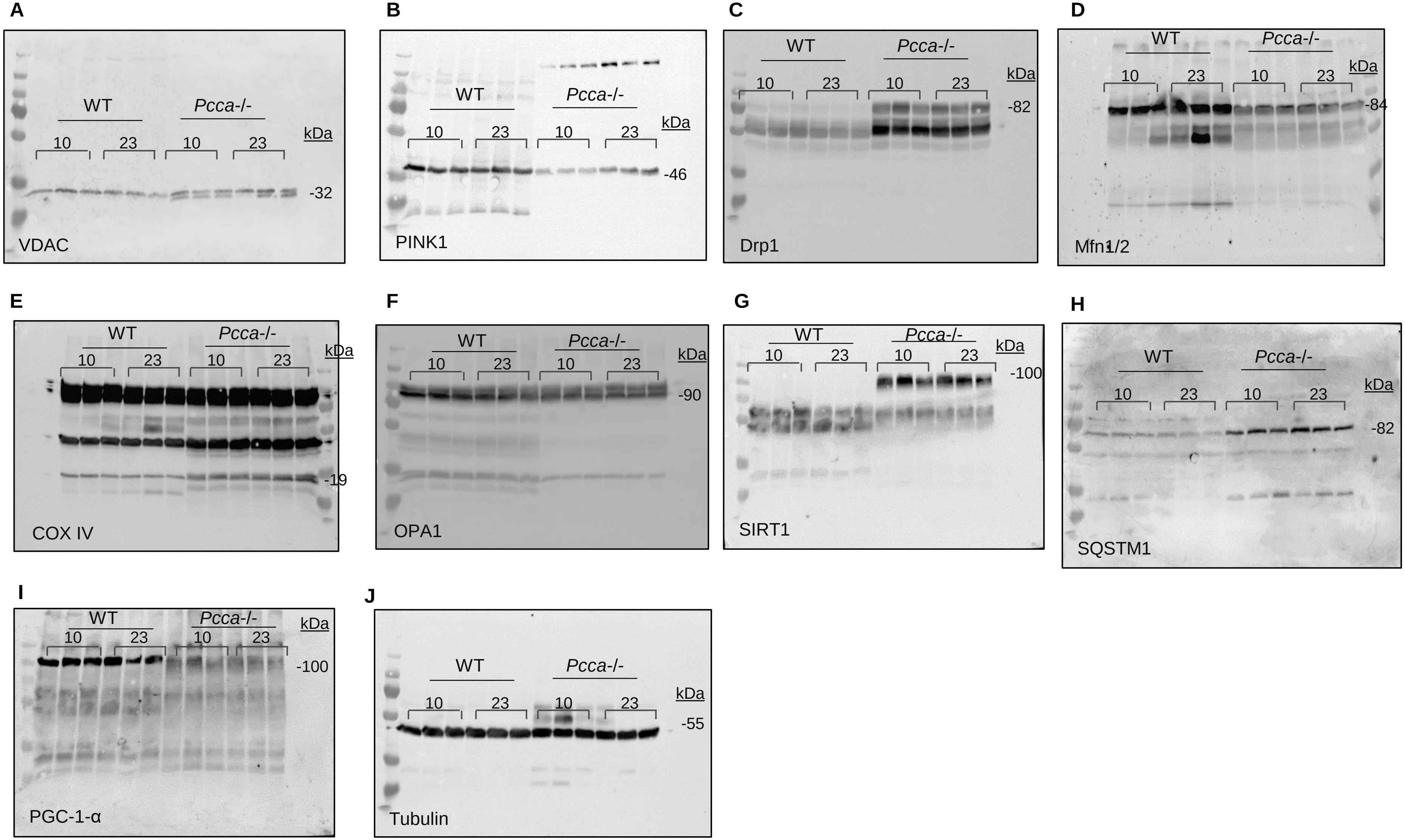

Supplementary Material

(A-J) representative uncropped and unprocessed versions of immuno-blots presented in the study.
