# Supplementary material for: Renal phenotyping in a hypomorphic murine model of propionic aciduria reveals common pathomechanisms in organic acidurias
Source: Sci Rep. 2024 Dec 16;14:30478. doi: 10.1038/s41598-024-79572-z (PMC11649940; doi:10.1038/s41598-024-79572-z)
Supplement: Supplementary file 4 — Supplementary Information 4. [file 41598_2024_79572_MOESM4_ESM.docx]

**Supplementary figure legends**

*Supplementary figure 1 Metabolic profiling of Pcca^-/-^(A138T) mouse kidneys*

Quantitative LC-MS/MS analysis of intermediate metabolites in 23-week-old WT and *Pcca^-/-^(A138T)* mouse kidneys. **(A)** Disease-associated metabolites. Lac: lactate, MC: Methylcitrate, MMA: Methylmalonic acid, Prop: propionate. **(B)** TCA cycle metabolites: Abbreviation of metabolites: α-KG: α-ketoglutarate, Cit: citrate, Eth: ethylmalonate, Ita: itaconate, Mal: malate, Malo: malonate, Suc: succinate. **(C)** Acylcarnitine profile of 23 week old WT and *Pcca^-/-^(A138T)* mouse kidneys. The acylcarnitines were summed up by the respective acyl-group chain-length: C0: free carnitine, C3: propionylcarnitine, SC: short-chain acylcarnitine (C2-C5), MC: medium-chain acylcarnitine (C6-C10), LC: long-chain acylcarnitine (C12-C18). Data normalized to WT. n=4 per group. **(D)** Cluster of medium-chain acylcarnitines (C8, C10) degraded by medium-chain acyl-CoA dehydrogenase and long-chain acylcarnitines (C14:1, C14:2) degraded by very long-chain acyl-CoA dehydrogenase. Data normalized to WT. n=4 per group.

*Supplementary figure 2 Amino acid profile in Pcca^-/-^ mouse kidneys*

Quantitative LC-MS/MS analysis of the amino acid profile in 23 week old WT and *Pcca^-/-^(A138T)* mouse kidneys. **(A)** Disease-associated amino acids. Gly: glycine, Ile/Leu: isoleucine/leucine, Met: methionine, Thr: threonine, Val: valine. **(B)** Disease-associated amino acid ratios. Ala/Lys: alanine/lysine, Ala/(Phe+Tyr): alanine/phenylalanine+tyrosine. **(C)** Complete amino acid profile: Ala: alanine, Arg: arginine, Asn: asparagine, Asp: aspartate, GABA: gamma-aminoisobutyric acid, Gln: glutamate, Glu: glutamine, Hist: histidine, Lys: lysine, Phe: phenylalanine, Pro: proline, Ser: serine, Tyr: tyrosine, Tryp: tryptophan. Data normalized to WT. n=4 per group.

*Supplementary figure 3 Expression profiles of mitochondrial markers from different compartments*

## RT-qPCR analysis of mitochondrial markers from different compartments in kidney extracts of 4-, 23- and 40-week-old WT and *Pcca^-/-^(A138T)* mice. n=3 per group, n=2 for WT 40-week-old mice. **(A)** Transcript levels of succinate dehydrogenase (*Sdh*, inner mitochondrial membrane). **(B)** Transcript levels of cytochrome c reductase (*Cycs*, intermembrane space). **(C)** Transcript levels of pyruvate dehydrogenase E1 subunit alpha 1 (*Pdha1*, mitochondrial matrix).
